# Crosstalk between cellular compartments protects against proteotoxicity and extends lifespan

**DOI:** 10.1038/srep28751

**Published:** 2016-06-27

**Authors:** Matea Perić, Peter Bou Dib, Sven Dennerlein, Marina Musa, Marina Rudan, Anita Lovrić, Andrea Nikolić, Ana Šarić, Sandra Sobočanec, Željka Mačak, Nuno Raimundo, Anita Kriško

**Affiliations:** 1 Mediterranean Institute for Life Sciences – MedILS, Meštrovićevo šetalište 45, 21000 Split, Croatia; 2 Universitätsmedizin Göttingen, Institut für Zellbiochemie, Humboldtallee 23, D-37073 Göttingen, Germany; 3 Division of Molecular Medicine, Ruđer Bošković Institute, Bijenička 54, 10000 Zagreb, Croatia

## Abstract

In cells living under optimal conditions, protein folding defects are usually prevented by the action of chaperones. Here, we investigate the cell-wide consequences of loss of chaperone function in cytosol, mitochondria or the endoplasmic reticulum (ER) in budding yeast. We find that the decline in chaperone activity in each compartment results in loss of respiration, demonstrating the dependence of mitochondrial activity on cell-wide proteostasis. Furthermore, each chaperone deficiency triggers a response, presumably via the communication among the folding environments of distinct cellular compartments, termed here the cross-organelle stress response (CORE). The proposed CORE pathway encompasses activation of protein conformational maintenance machineries, antioxidant enzymes, and metabolic changes simultaneously in the cytosol, mitochondria, and the ER. CORE induction extends replicative and chronological lifespan in budding yeast, highlighting its protective role against moderate proteotoxicity and its consequences such as the decline in respiration. Our findings accentuate that organelles do not function in isolation, but are integrated in a functional crosstalk, while also highlighting the importance of organelle communication in aging and age-related diseases.

Cells have acquired multiple mechanisms for the maintenance of protein structure and function. This implies an activity that would enable thousands of cellular proteins to fold, correctly and efficiently, under both optimal and challenging conditions^1^. Molecular chaperones, including the heat-shock proteins (Hsps), are ubiquitously present cellular proteins, which display a wide spectrum of folding-oriented activities, coping with regular protein folding events as well as stress-induced protein misfolding^2^. Naturally, such protein homeostasis (proteostasis) may decline in performance, as seen in numerous diseases and aging. As a result of such decline, proteins cannot maintain their native fold, perform their function and, consequently, tend to form non-soluble cytotoxic aggregates^3^.

In compartmentalized eukaryotic cells, several independent pathways exist that ensure the integrity of the protein-folding environments in the cytosol, the endoplasmic reticulum (ER), and the mitochondria. In addition to the cytosolic heat shock response^4^, several main pathways deal with failure of proteostasis in specific compartments. The endoplasmic reticulum (ER) serves to properly fold and process proteins destined for secretion and membranes. Conditions that disrupt the ER function cause an accumulation of misfolded proteins in the ER lumen, whose turnover is usually mediated by ER-associated degradation^5^,^6^, with a task to alleviate this stress and restore ER homeostasis, promoting cell survival and adaptation^7^,^8^. Perturbations in the mitochondrial folding environment trigger a similar response in the mitochondrial lumen, thus activating nuclear-encoded chaperones dedicated to restore proteostasis in this organelle^9^. All three compartments require assistance in early folding events as well as management of downstream misfolding. The current knowledge posits that misfolded protein stress is sensed in a compartment-specific manner to induce the expression of compartment-specific chaperones.

In this study we addressed the persisting question of cell-wide consequences of proteostasis failure in specific cellular compartments. We monitored the effect of loss-of-function of cytosolic, mitochondrial, and ER chaperones, each involved in protein input into different organelles, as well as protein folding. Our results show that the loss of each studied chaperone, regardless of the compartment of its residence and activity, induces a common cross-organelle response (CORE) that includes protein maintenance and antioxidant responses in the cytosol, mitochondria and the ER, without activating any of the canonical stress response pathways. The observed striking decrease in respiration, without an obvious defect in the respiratory chain, suggests an active suppression of respiration, highlighting the importance of cell-wide protein maintenance. The most prominent metabolic feature of the chaperone deficient strains is the provision of metabolic intermediates for anabolic biosynthesis - a feature likely to be important for cells coping with mild stress. Our observation that the induction of such cross organelle stress response extends both replicative and chronological lifespan in budding yeast implies CORE’s protective role against mild proteotoxicity. This study uncovers a crosstalk between distinct subcellular compartments as yet another stress response mechanism whose regulation requires further elucidation.

## Results

### Proteotoxicity in any cellular compartment leads to a decline in respiration

**Figure 1:**
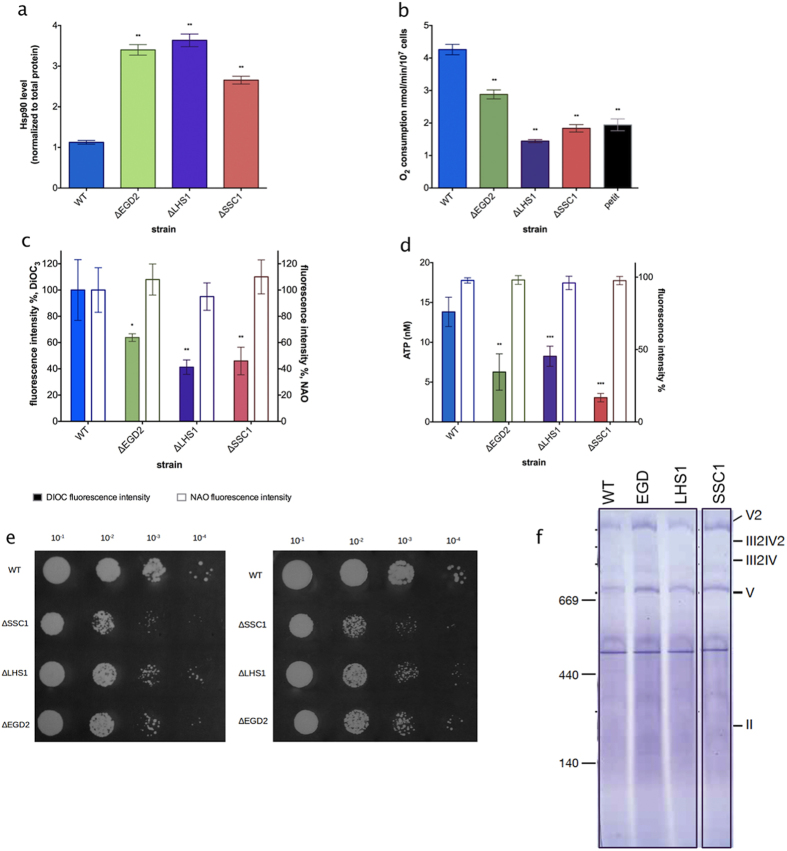
Chaperone deletion mutants are characterized by increased proteotoxicity and repressed mitochondrial activity. **(a)** Hsp90 levels are increased in the chaperone deficient mutants relative to the control. **p < 0.01 (ANOVA plus post hoc). **(b)** Oxygen consumption rates are decreased in the chaperone deficient mutants. Oxygen consumption is measured polarographically in the YPD growth medium supplemented with 2% glucose at 30 °C. **(c)** Mitochondrial membrane potential-related fluorescence via DiOC6(3) is decreased. After collapsing the ΔΨm by 10 min preincubation with 100 μM CCCP and 5 μg/mL antimycin, signal intensity decreased, indicating mitochondrial membrane depolarization. Mitochondrial biomass is unchanged in the chaperone deficient mutants. The NAO fluorescent signal of energized mitochondria was collected in the appropriate channel following staining. **(d)** ATP levels are decreased in the chaperone deficient mutants, without change in ROS levels. Data are represented as mean ± SD from 3 independent cultures, each measured in duplicate. ***p < 0.001; **p < 0.01 (ANOVA plus post hoc). **(e)** Drop test results display no difference in growth on YPEG medium between the studied strains. Growth on YPEG plates is preceded by growth in the same liquid medium. Left panel displays results of the exponentially growing cells, and the right one of the stationary cells. **(f)** Blue Native gels showing the level of Complexes II, IV and V in the wild type and the chaperone deficient strains.

In order to induce protein stress in several different cellular compartments, we independently deleted a gene copy of three protein chaperones: cytosolic nascent polypeptide associated complex (NAC, EGD2), HSP70 chaperone from the endoplasmic reticulum (erHSP70, LHS1), and mitochondrial HSP70 (mtHSP70, SSC1). Interestingly, regardless of the compartment of protein stress origin, all mutants experience the increase in cytosolic Hsp90 levels, relative to the WT (Fig. 1a, Supplementary Fig. S1).

At this point, we were interested to characterize a cellular process fundamental for proper cell functioning. Therefore, we set out to determine the impact of proteostasis failure in different compartments on mitochondrial respiration. First, we measured O_2_ consumption in yeast mid-log phase in 2%-glucose media, mimicking flask culture conditions. The deficiency of each chaperone led to a significant decrease in O_2_ consumption in comparison to the WT strain (Fig. 1b), rendering it similar to the respiration-deficient petite strain (included as a negative control). Furthermore, DiOC6(3) stained chaperone deficient strains displayed a significant decrease in fluorescent intensity, relative to the WT strain (Fig. 1c), indicating a decline of mitochondrial membrane potential (MMP). Since O_2_ consumption can also decline as a result of decreased mitochondrial mass, we used a flow cytometry based assay where nonyl acridine orange (NAO) fluorescence is indicative of cardiolipin content, in turn correlating with the mitochondrial mass. No significant difference in NAO fluorescence intensity was observed among the studied strains, indicating that there has been no change in mitochondrial mass (Fig. 1c). This observation is further corroborated by the decreased levels of ATP in the deletion mutants compared to the control, accompanied by unchanged levels of ROS (Fig. 1d).

Next, we tested the possibility that the observed decline in respiration is a result of a chaperone deficiency-related respiratory chain (RC) defect. We therefore evaluated the growth of the wild type and the mutant strains in the YPEG medium (3% ethanol, 3% glycerol), where only respiration competent cells are able to grow, while respiration defective cells are selected against. We observed no significant difference in growth between the wild type and the mutant strains in exponential or stationary phase (Fig. 1e), prompting us to conclude that the observed decrease in respiration is not a result of defective respiratory chain complexes. To further investigate the steady state levels of the mitochondrial respiratory chain complexes, mitochondria were solubilized in the mild detergent Digitonin and subjected to the Blue Native PAGE analysis. We found no significant difference in RC composition between the strains (Fig. 1f).

### Localized proteotoxicity leads to a cell-wide response

**Figure 2:**
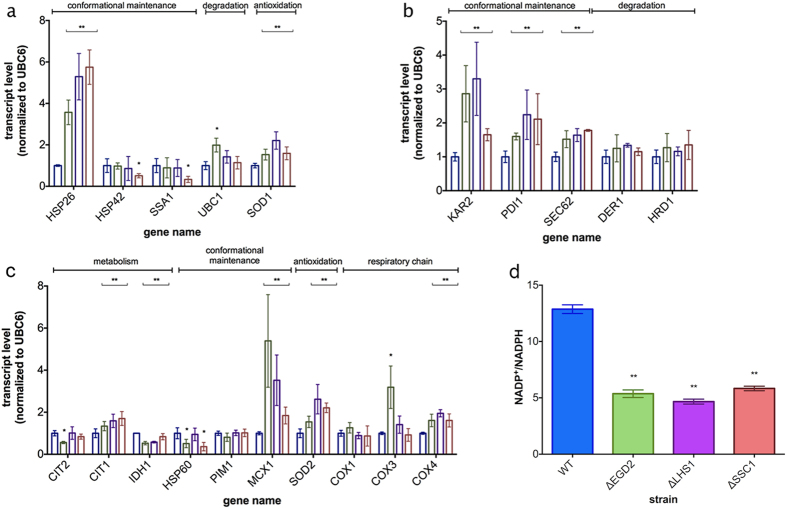
Localized chaperone deficiency does not activate any of the key cellular stress responses. Transcript levels of **(a)** cytosolic, **(b)** ER and **(c)** mitochondrial target genes in the chaperone deficient strains, compared to the WT. UBC6 was used as a control. The measurement was performed in biological and technical triplicate. **(d)** NADP/NADPH ratio is decreased in the chaperone deficient mutants. Data are represented as mean ± SD from 3 independent cultures, each measured in duplicate. ***p < 0.001; **p < 0.01 (ANOVA plus post hoc).

The observed decline in respiration motivated us to measure trends in gene expression using qPCR in several canonical stress response pathways related to all three relevant compartments: mitochondrial retrograde response, ERAD, and cytosolic heat shock response (Fig. 2). The most prominent feature of the chaperone deficient strains is the upregulation of the cytosolic HSP26, normally in charge of targeting proteins to either aggregation or refolding during heat shock (Fig. 2a). Interestingly, the level of HSP42, a small HS protein of similar function, remains unchanged, as well as Hsp70 (SSA1), usually a hallmark of cytosolic HS response activation.

Similarly, in the case of typical ERAD components, only proteins involved in the ER luminal folding displayed an increase in expression (KAR2, PDI1 and SEC62) while protein degradation remained largely unaffected (DER1, HRD1, UBC1) (Fig. 2b). The difference is most prominent in the case of KAR2 (2–4 fold increase in the chaperone deficient strains), while PDI1 and SEC62 display a smaller but significant increase of 1.5–2 fold (Fig. 2b).

Moreover, antioxidant protection was slightly upregulated (1.5–2.5 fold) in both the cytosol (SOD1) and the mitochondria (SOD2) (Fig. 2a,c), indicating leakage of ROS into the cytosol, probably due to changes in the RC activity or the existence of a feed-forward loop preventing the dysfunctional mitochondria from putting the cell under oxidative stress. An imbalance in the expression of the complex IV subunits could cause such a change in the RC activity: while COX4 expression was increased (2-fold in average), the levels of COX1 and COX3 remain unchanged (Fig. 2c).

### The cell-wide response includes an increase in the cytosolic NADPH reducing power

Since retrograde response, a cellular response to mitochondrial dysfunction, may have an effect similar to the one observed in the mutant strains, we wanted to rule out its involvement. Considering that the levels of peroxisomal citrate synthase involved in glyoxylate cycle (CIT2) were unchanged (Fig. 2c), we concluded that there is no evidence of retrograde response involvement. However, an interesting metabolic feature is put forward: the upregulation (1.5–2 fold) of the mitochondrial citrate synthase (CIT1) and the downregulation (app. 0.5–0.7 fold) of isocitrate dehydrogenase (IDH1) likely leads to accumulation of citrate. Further, evidence of activation of mtUPR (HSP60) and degradation by mitochondrial Lon protease (PIM1) was absent. However, MCX1, a mitochondrial chaperone with poorly described function was upregulated (Fig. 2c).

In order to characterize the potential consequences of the changes in the TCA cycle enzyme levels, we measured the NADP^+^/NADPH ratio, whose decrease usually points towards increase in the reducing power in the cytosol. Even though certain variability is present, NADP^+^/NADPH ratio is significantly decreased in the chaperone deficient strains relative to the control (Fig. 2d).

Taken together, our data suggest that localized proteotoxicity induces a response across the entire cell, one that includes metabolic changes and activation of folding maintenance machinery in multiple compartments. We therefore termed the response cross-organelle stress response (CORE).

### Localized proteotoxicity leads to mitochondrial fragmentation

**Figure 3:**
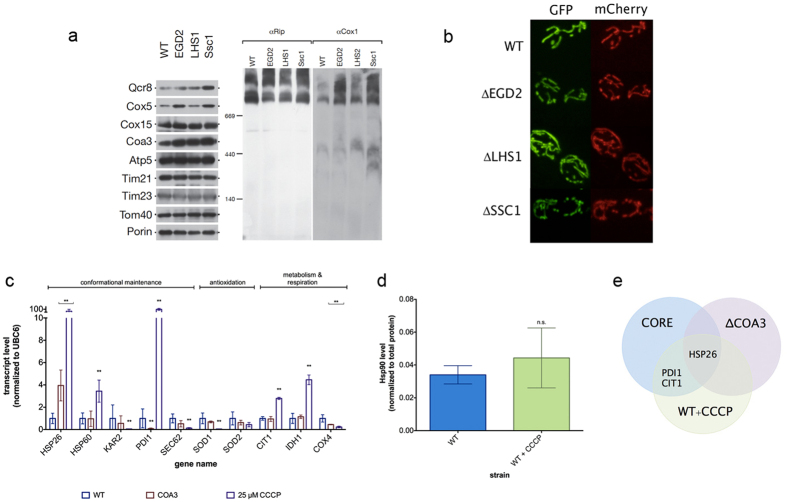
CORE pathway is partially a response to respiration decline. **(a)** Western blot analysis of several respiratory chain components. Porin was used as a loading control. **(b)** Representative images of cells with visualized mitochondria via preCOX4-mCherry and preSU9-GFP. **(c)** CORE pathway transcript level in the Coa3-null mutant as well as the wild type treated with 25 μM CCCP. **(d)** Hsp90 levels are unaffected by the CCCP treatment in the wild type strain. Data are represented as mean ± SD from 3 independent cultures, each measured in duplicate. ***p < 0.001; **p < 0.01 (ANOVA plus post hoc). **(e)** Venn diagram of the overlap between the responses in chaperone deficient strains, Coa3-null mutants, and the CCCP-treated wild type cells.

Further, we set to determine the levels of respiratory chain components by Western blot (Fig. 3a, Supplementary Fig. S2). We tested the steady state levels of Qcr8, a subunit of Complex III, the Complex IV components Cox1 and Cox5, the Complex IV assembly factors Coa3 and Cox15, as well as the Complex V subunit Atp5. While Atp5 and Cox15 showed no change in its level upon chaperone deficiency, the integral subunits of Complex III and Complex IV components displayed a different behavior (Fig. 3a, Supplementary Fig. S2). Although only 2-fold in average, Qcr8 and Cox5 levels showed an increase only in the EGD2 and SSC1 deficient strains, while Cox1 and Coa3 level increased in all chaperone deficient mutants. In addition, we tested the steady state levels of the translocase of the outer mitochondrial membrane (TOM) Tom40, and subunits of the translocase of the inner mitochondrial (TIM) membrane Tim21 and Tim23 (Fig. 3a, Supplementary Fig. S2). We found that none of the components displayed any variation between the wild type and the mutants. Furthermore, no clear difference was observed after decoration against the ubiquinol-cytochrome-c reductase protein (Rip1).

In order to visualize mitochondrial morphology, as well as protein import into mitochondria, we employed MitoLoc plasmid, which allows localization analysis of differentially imported fluorescent marker proteins^10^. Briefly, GFP is fused to the fungal mitochondrial localization signal of the F_0_-ATPase subunit 9 (preSU9) of *Neurospora crassa*, whose signal is independent of the mitochondrial membrane potential (MMP), while mCherry is fused to the N-terminal localization sequence of cytochrome C oxidase 4 (COX4), and is imported into mitochondria proportional to the MMP. We found that mitochondria in chaperone deficient strains are smaller and more round than the tubular mitochondria found in WT, which is indicative of mitochondrial fragmentation (Fig. 3b). More specifically, analysis of the mitochondrial volume using MitoLoc ImageJ plugin revealed a distribution shift towards smaller volumes in chaperone deficient mutants relative to the wild type. No significant increase has been detected in the fraction of cells with accumulation of mCherry-Cox4 in the cytosol, suggesting that Cox4 is successfully translocated into the inner mitochondrial membrane (Supplementary Fig. S3). Moreover, both fluorescent signals always co-localized in a similar fraction of cells in both the wild type and the chaperone deficient mutants (Fig. 3, Supplementary Fig. S3). These results suggest that defective protein import into the mitochondria of the chaperone deficient mutants, which could explain the partial upregulation of cytosolic chaperones, is an unlikely scenario.

However, we have considered this possibility further. We thus measured the expression levels of the likely components of the CORE pathway using qPCR in: (i) respiration defective mutant with inactivated COA3 gene, encoding for an assembly factor of the Complex IV, and (ii) the wild type strain treated with CCCP, thus abolishing the obligatory linkage between the respiratory chain and the phosphorylation system.

In the case of COA3-null mutant, only HSP26 upregulation was in common with the proposed CORE pathway (Fig. 3c). Furthermore, uncoupling of the respiratory chain with CCCP yielded a response partially overlapping with the response observed in chaperone deficient mutants. It included upregulation of Hsp26 chaperone and the protein disulfide isomerase, PDI1. Additionally, CCCP treatment results in citrate synthase (CIT1) upregulation, albeit without accompanying downregulation of IDH1 as in the chaperone deficient strains (Fig. 3c). However, CCCP treatment yielded other changes, which are not observed in the framework of the proposed CORE pathway: increase in the expression level of the mitochondrial Hsp60, a sharp decrease in the expression level of other ERAD components, SEC62 and KAR2, as well as in SOD1. CIT1 and IDH1 experienced an increase in the expression level, unlike in the context of the proposed CORE pathway (Fig. 3c). In addition, CCCP treatment of the wild type strain did not affect the Hsp90 level (Fig. 3d, Supplementary Fig. S4). These results confirm that the overlap between the CORE pathway and RC uncoupling or an RC defect is minor and that the CORE pathway is unlikely a response to defective protein import and only in small part a response to the decline in respiration.

### CORE pathway partially relies on Hsf1 activation

**Figure 4:**
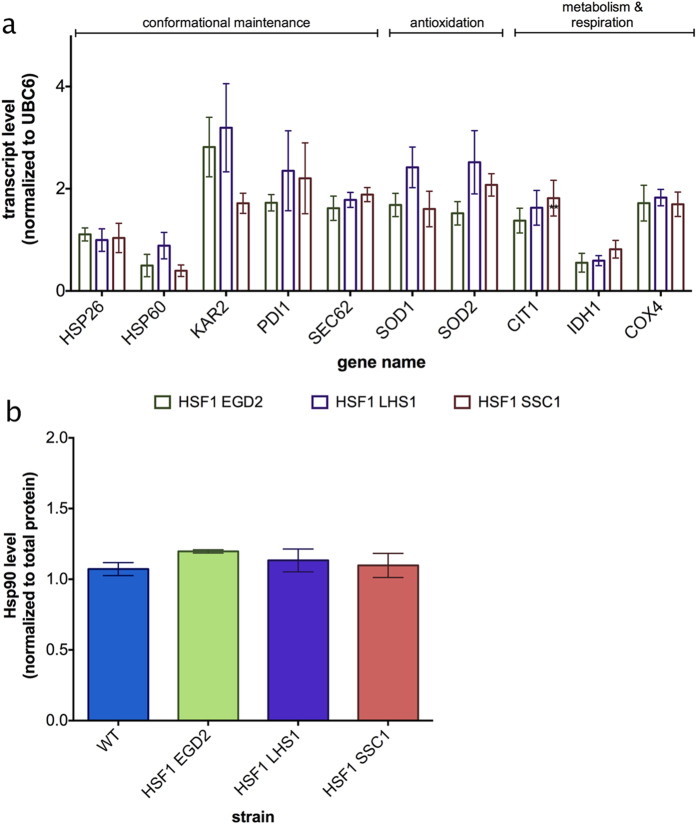
Hsf1 regulates a part of the CORE pathway. **(a)** Transcript levels of genes proposed to be involved in the CORE pathway. UBC6 was used as a control. The measurement was performed in biological and technical triplicate. **(b)** Hsp90 levels remain unaffected by the deletion of studied chaperones in the absence of Hsf1. Data are represented as mean ± SD from 3 independent cultures, each measured in duplicate. ***p < 0.001; **p < 0.01 (ANOVA plus post hoc).

In order to investigate the role of Hsf1 in the regulation of the proposed pathway, we measured the expression levels of the likely constituents in the double deletions of Hsf1 in combination with each studied chaperone using qPCR. We found that in the absence of Hsf1, chaperone deletions did not result in the upregulation of Hsp26 (Fig. 4a) and Hsp90 (Fig. 4b, Supplementary Fig. S5). On the other hand, the oxygen consumption (Supplementary Fig. S5) and the metabolic features of the proposed CORE pathway remained at the level observed in the chaperone deficient strains (Fig. 4a), suggesting that this part of the pathway is regulated in a different manner.

### CORE pathway activation extends replicative and chronological lifespan

**Figure 5:**
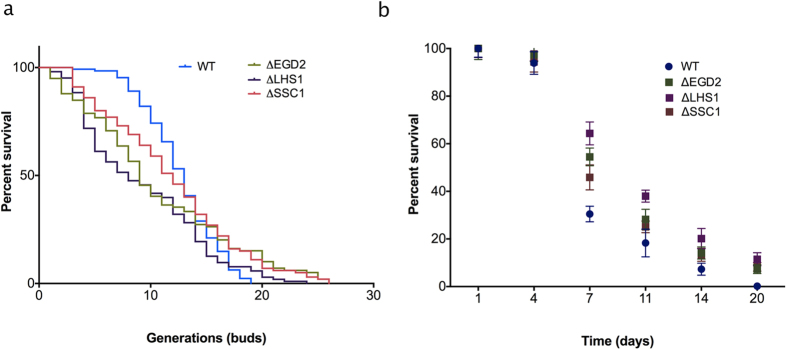
Replicative and chronological lifespan are extended in the chaperone deletion mutants. **(a)** Replicative lifespan is assessed by micromanipulation. The number of cells is 104, 117, 115, and 121 for the wild type, ∆EGD2, ∆LHS1 and ∆SSC1, respectively. The data shown are pooled from 2 independent experiments for each strain. Significance of the results was tested with log-rank test, p-values < 0.05. **(b)** Chronological lifespan is assessed by measuring survival in post-mitotic yeast culture every three days by plating. The results represent mean and standard deviation of three independent experiments.

Cellular stress responses, when countering mild stress, often have overall beneficial effects on a cell, rather than providing merely a survival route. Therefore, we have set out to measure the replicative lifespan (RLS) of the studied chaperone deficient mutants. RLS is measured as the maximum number of generations that each mother cell goes through before the onset of senescence. The control strain produced a maximum of 19 buds during its RLS, which corresponds to the expected value for this strain (Fig. 5a). The largest effect on RLS with a 40% lifespan extension, in comparison to the control, resulted from the deletions of EGD2, encoding a subunit of the nascent polypeptide associated complex (NAC), as well as SSC1, mtHSP70. Finally, the deletion of LHS1, erHsp70, resulted in 30% lifespan extension relative to the control. Furthermore, we monitored the chronological lifespan (CLS) of the studied strains, measured as the mean and maximum survival time of non-dividing yeast populations (Fig. 5b). As with the replicative lifespan, we found that the chronological lifespan was extended in all chaperone deficient mutants, with the largest effect in the deletion of LHS1 (app 40%), followed by the deletion of EGD2 with 25% extension (Fig. 5b). As in the case of RLS, the smallest effect was observed in the deletion of the SSC1, with only 15% extension (Fig. 5b).

In summary, both RLS and CLS extensions confirm the beneficial role of the CORE pathway in the context of cellular aging.

## Discussion

Here we present the existence of a novel pathway, termed the cross-organelle response (CORE), which enables cells to deal with mild proteotoxic stress, thereby extending their lifespan.

The activation of known cellular stress responses is preceded by the detection of misfolded proteins and is largely confined to the compartment under stress. The novelty of the CORE stress response is that the unfavorable conditions in individual folding environments of mitochondria, ER, and cytosol are translated into other compartments generating a cell-wide, cross-organelle response. While it was already reported that mild cytosolic protein stress could exert a protective effect in both ER and cytosol^11^, the existence of folding environment communication among these compartments, and the uniformity of the cell-wide stress response, was unknown.

It is a feature of CORE that, regardless of the compartment in which the chaperone is deficient, the stress response seems to be cell-wide and unique in all studied strains. The response consists of changes in two groups of genes: (i) cellular maintenance, and (ii) metabolic changes, including the decline in respiration.

**Figure 6:**
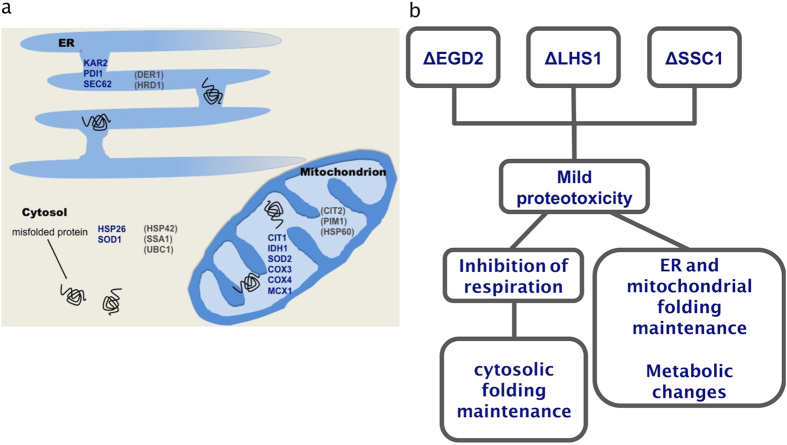
The CORE pathway activates genes simultaneously in multiple compartments. **(a)** A schematic presentation of the genes (in blue) with modified gene expression in the context of CORE. In brackets are the genes associated to compartment-specific stress response, but unchanged in the chaperone deletion mutants. **(b)** The proposed sequence of events in the framework of CORE.

One of the hallmarks of the CORE pathway is the upregulation of multiple conformational maintenance machineries (Fig. 6a). The Hsf1-dependent upregulation of Hsp26 and Hsp90 emphasizes that the cells rely on efficient elimination of misfolded proteins through both their aggregation and refolding^12^,^13^,^14^. Like many other small heat shock proteins, Hsp26 efficiently binds its substrates without refolding^15^, and is characterized by its ability to transfer substrates to Hsp70^16^. However, Hsp70 upregulation - usually also a hallmark of the heat shock response (HSR) - is absent in CORE, thus excluding the canonical activation of HSR. Moreover, several analyzed members of the ERAD pathway show that folding maintenance (KAR2), and to a lesser extent translocation into ER lumen (SEC62), and oxidative folding (PDI1) are upregulated, unlike the components responsible for degradation (DER1, HRD1, UBC1). Similarly, mitochondria rely on upregulation of Mcx1, also a member of the conformational maintenance machinery. Thus, in the context of the CORE pathway, the cell seems to invest energy to protect the folding environments of the cytosol, mitochondria and the ER, all of which are sites of cellular protein synthesis and/or folding events (Fig. 6a). The observed moderate upregulation of SOD1 and SOD2, likely has a role to insure against an increase in ROS that can inflict irreversible oxidative damage to less-than-perfectly folded proteins and prevent their eventual refolding^17^,^18^.

Moreover, CIT1 upregulation, combined with downregulation of IDH1, an enzyme catalyzing the subsequent step of the TCA cycle, is the most prominent metabolic feature of the studied mutants, likely leading to citrate accumulation. A dedicated mitochondrial carrier for citrate (from mitochondria to cytosol) and oxaloglutarate (from cytosol to mitochondria) enables transport of citrate from the mitochondrial matrix to the cytosol^19^. Generally, the main purpose of this transport is to increase the NADPH reducing power in the cytosol (required for biosynthetic and antioxidant reactions) and probably to act as a key component of the citrate-oxaloglutarate NADPH redox shuttle between mitochondria and cytosol^19^. Together with the observed changes in the TCA cycle of the chaperone deficient strains, the decrease in the NADP^+^/NADPH ratio strongly supports this scenario. In this way, one of the main TCA cycle functions comes to the forefront by providing metabolic intermediates for anabolic biosynthesis, a feature likely to be important in cells coping with mild stress^20^.

The results presented here imply that impeccable proteostasis across cellular compartments is essential for a functional respiratory chain: one of the outstanding features of the chaperone deficient mutants is the decline in respiration. Prompted by the observed decrease of the MMP, we tested for the induction of the retrograde response^21^, a pathway through which mitochondria communicate their MMP decline to the nucleus and influence many cellular functions^22^. However, the induction of CIT2, encoding the peroxisomal citrate synthase, commonly a hallmark of activated retrograde response^22^, is absent in our mutants. This led us to speculate that the decline in MMP is not a result of proteostasis failure, but rather, an active downstream consequence of the CORE pathway activation (Fig. 6b). For instance, a cell undergoing protein stress may actively decrease mitochondrial function in order to ‘silence’ the major source of ROS, since increased ROS levels combined with proteotoxic stress would have a devastating effect on cellular functioning^18^.

The existing literature on the chaperone deficiencies studied here offers potential interpretation of the observed phenotype: Egd2 and Lhs1 decline causes slight defects in protein import into the mitochondria^23^,^24^ and the ER^25^, as well as folding impairment in the ER lumen^25^. Moreover, Ssc1 has been proven essential for protein translocation into the mitochondrial matrix^26^. Clearly, the deficiencies of each of these chaperones may cause protein accumulation in the cytosol and the decline in mitochondrial activity. Even though we have not observed cytosolic accumulation of preCox4-mCherry, whose import into the mitochondrial inner membrane is MMP-dependant, we cannot exclude the possibility that the organelle import of specific proteins has indeed failed in the chaperone deficient strains. Therefore, we studied the extent to which CORE may be a response to such protein accumulation by hindering the protein import into mitochondria in two independent ways: while CCCP treatment results in decreased level of cellular ATP, thereby affecting protein translocation into both ER and mitochondria, protein folding across the cell, as well as protein degradation, COA3-null mutant mainly results in defective mitochondrial import due to loss of the inner membrane potential.

The results obtained on the COA3-null mutant, and the wild type treated with CCCP, demonstrate two important points:

1. CORE pathway is largely not a response to the decline in mitochondrial function (MMP), or failure of the RC. We determined that only the upregulation of Hsp26 and PDI1 have likely resulted from the failure in protein targeting into organelles, i.e. decline in the MMP. Other features seem to be specific to the cross-organelle response triggered by mild proteotoxicity in individual compartments and by inter-organelle communication.
2. The metabolic changes characteristic of the chaperone deficient mutants, not observed in neither CCCP treatment, nor in the COA3-null mutant, may have a role to mitigate the proteotoxicity by aiding cellular adaptation to requirements of coping with stress.

In summary, these results suggest a sequence of events, all triggered by chaperone deficiency and proteostasis failure in each compartment (Fig. 6b). Mild proteotoxic stress likely initiates the changes leading to a decline in the MMP and oxygen consumption, while also triggering changes in cellular metabolic activity. The decline in MMP causes failure of protein import into organelles and gives rise to upregulation of Hsp26 and Hsp90, via Hsf1, and PDI1. The role and regulation of the remaining constituents of the CORE pathway (ERAD and the metabolic changes) will be a subject of future studies.

The cellular responses to stress are known to be tightly related to aging^3^. Here, we see both replicative and chronological lifespan extension in budding yeast, despite proteostasis failure, and likely related to the decline in mitochondrial activity; mild inhibition of mitochondrial activity has already been shown to extend lifespan across species^27^,^28^,^29^. Interestingly, the ‘kinetics’ of replicative aging differ significantly in the studied chaperone deficient mutants relative to the wild type, displaying an initial steep decrease in replicative survival followed by an extended late life stage. Generally, all strains displayed a certain distribution in lifespans of individual cells. Here, the wild type population goes through the maximum number of 19 divisions, while in the chaperone deficient mutants 7–10% of the population goes through more than 20 divisions, with the maximum of ≈25 divisions. On the other hand, chronological aging is assessed in cells in stationary growth phase, in which carbon sources are exhausted, yeast budding rates are negligible, and mitochondria are the main source of cell ATP production^30^. Common features of the mechanisms that regulate replicative and chronological lifespan are poorly understood. However, studies suggest that chronological and replicative lifespans are regulated by overlapping but distinct mechanisms^30^. The lifespan extensions are likely induced by the CORE pathway activation in response to the proteotoxic stress. In general, the effects of stress on cells seem to be dose-dependent: mild stress induces stress tolerance and extends the lifespan (in aging research known as hormesis^31^,^32^), whereas excessive stress aggravates the aging process.

The questions persist how the information on the folding environment status is communicated between the organelles and why none of the canonical stress responses have been activated by the deficiency of the three studied chaperones. At this point, we can only speculate that due to redundancy with other chaperones in each compartment, the cell perceives the absence of each of the three chaperones as mild proteotoxic stress. Therefore, specific signals needed to activate some of the canonical stress response pathways are likely to be missing during CORE, while the nature of signals generated to communicate the status of folding environment between cellular organelles will be a subject of further research.

In summary, we show that independent perturbation of proteostasis in different cellular compartments results in induction of a unique cross-organelle stress response that culminates in the increase of yeast RLS and CLS. These results underscore the relevance of intracellular communication across different compartments to achieve coordinated signaling programs. This exemplifies that the crosstalk between organelles and pathways renders classical canonical pathways redundant and accentuates the need of studying cellular responses in a system-wide fashion.

## Methods

### Strains and growth conditions

Wild type *Saccharomyces cerevisiae* Y258 was used. WT Y258 and deletion strains of Lhs1, Ssc1, and Egd2, were purchased from Thermo Scientific (Dharmacon). It should be noted that each deletion strain had only one copy of the gene deleted, resulting in significantly decreased amount of each chaperone expression levels. Coa3-null mutant was constructed as described in Mick *et al*.^33^.

All strains were grown on YPD medium with 2% (w/v) glucose at 30 °C with shaking. All experiments were performed on cells from mid-exponential phase: cells were grown until OD 0.6–0.8, harvested by 5 min centrifugation at 4000 × g, washed and treated accordingly. In order to test the respiratory chain efficiency in the studied strains, they were grown in the YPEG medium containing 3% ethanol and 3% glycerol, at 30 °C with shaking. Both exponentially growing and stationary cells were serially diluted and 5 μL drops were plated onto YPEG agar plates. Growth was observed after 4 days. For the CCCP treatment, exponentially growing cells were exposed to the final concentration of 25 μM CCCP and incubated for 2 h at 30 °C with shaking.

### Replicative lifespan measurement

Replicative lifespan (RLS) for all strains was determined by micromanipulation. RLS measurement involves counting the number of daughters produced by individual mother cells. Cells were incubated at 30 °C on YPD plates for the duration of the experiment. Using a microdissection apparatus equipped microscope suitable for yeast (Singer Instruments), cells were transferred to defined places on the agar plates and virgin daughter cells were collected. Each cell was monitored continuously over several days every 60–90 min until all mother cells stopped budding. Total number of daughter cells was noted for each mother cell.

### Chronological lifespan measurement

All strains were grown until stationary phase in YPD medium at 30 °C with shaking, followed by cell pelleting at 4000 × g for 5 min. Cells were then resuspended in sterile deionized water and incubated at 30 °C with shaking. Every 3 days, cells were serially diluted and plated onto YPD plates in order to evaluate cells growth.

### Respiration measurement

Oxygen uptake was monitored polarographically with an oxygraph equipped with a Clark-type electrode (Oxygraph, Hansatech, Norfolk, UK). Cells were harvested during exponential growth phase, spun and resuspended in appropriate medium at the density of 30 × 10^6^ cells/mL. 500 μL of culture were transferred to an airtight 1.5 mL oxygraph chamber. Cells were assayed in conditions closely similar to the ones in a flask culture (30 °C and stirring). Oxygen content was monitored for at least 4 min. To ensure the observed oxygen consumption was due to the mitochondrial activity, complex III inhibitor antimycin (final concentration 10 μg/mL) was routinely added to the cultures and compared to the rate observed without antimycin.

### Flow cytometry

Flow cytometry was carried out on a Becton-Dickinson FACSCalibur model equipped with a 488 nm Argon laser and a 635 nm red diode laser.

### ROS measurement

Cells were incubated in the dark with 10 μg/μL 2′,7′-dichlorofluorescein diacetate (H2DCFDA, Sigma) for 120 min at 37 °C and subsequently analyzed on Becton-Dickinson FACSCalibur flow cytometer equipped with a 488 nm Argon laser and a 635 nm red diode laser. The fluorescence of 10,000 cells resulting from the intracellular conversion of non-fluorescent H2DCFDA into fluorescent 2′,7 -dichlorofluorescein (DCF) was measured in FL1 channel. The collected data was analyzed using FlowJo software version 7.2.5 for Microsoft (TreeStar, San Carlos, CA, USA) to determine the mean green fluorescence intensity after each treatment. The results are expressed as the mean fluorescence of the 10,000 cells.

### Assessment of Mitochondrial Membrane Potential

Variations of the mitochondrial transmembrane potential (ΔΨm) were studied using 3,3′-dihexyloxacarbocyanine iodide (DiOC6(3)). This cyanine cationic dye accumulates in the mitochondrial matrix directly under the influence of the ΔΨm^34^. Cells (1 × 10^6^/mL) were incubated in 1 mL culture medium containing 40 nM DiOC6(3) for 30 min in the dark at 30 °C with constant shaking. DiOC6(3) membrane potential-related fluorescence was recorded using FL1-height. A total of 10,000 cells were analyzed for each curve. The collected data was analyzed using FlowJo software version 7.2.5 to determine the mean green fluorescence intensity after each treatment. The results are expressed as a percentage of mean fluorescence of the control strain. For negative control, in each experiment, aliquots of cells were used to achieve the collapse of the mitochondrial membrane potential by preincubation with carbonyl-cyanide 4-(trifluoromethoxy)- phenylhydrazone (FCCP, Sigma) and antimycin (Sigma) at 100 μM and 5 μg/mL, respectively, 10 min before fluorescent dye staining.

As a measure of mitochondrial mass, we used 10-N-*Nonyl acridine orange* (NAO), a dye that binds to cardiolipin, a phospholipid specifically present on the mitochondrial membrane^34^. The cells (1 × 10^6^/mL) were incubated in 1 mL culture medium containing 100 nM NAO for 30 min in the dark at 30 °C with constant shaking, followed by analysis on FACSCalibur flow cytometer with the same photomultiplier settings as used for DiOC6(3).

### Western blot detection of Hsp90

Cells were incubated in 1 mL lysis buffer (PBS, 60U zymolyase, protease inhibitor cocktail) for 1 hr at 37 °C. Cell pellet was resuspended in spheroplast lysis buffer (1 mL buffer per 0.5 g of cell pellet; 0.6 M Sorbitol, 10 mM Tris-HCl pH 7.4, 1 mM PMSF). Cells were vigorously vortexed for 1 min, and left on ice for 30 min, with occasional vortexing. After centrifugation, supernatant was collected, and protein concentration was measured using Bradford reagent (Sigma).

50 μg of protein was mixed with Laemmli sample buffer (10% SDS, 20% glycerol, 10 mM 2-mercaptoethanol, 0.05% bromophenol blue), heated to 95 °C for 5 min, and loaded onto two 7.5% SDS PAGE gel, 25 μg of protein each. Proteins from one gel were then transferred onto a nitrocellulose membrane at 200 mA for 1 hr, while the second gel was stained by Coomassie Brilliant Blue for 20 minutes followed by destaining.

For the determination of Hsp90 chaperone, the membrane was blocked with 5% milk in PBS containing 0.1% Tween-20 for 1 hr. Anti-Hsp90 antibody (1 mg/mL) was diluted in blocking buffer (1:2500, StressMarq Biosciences), and incubated overnight at 4 °C. Detection was done by horseradish peroxidase-conjugated goat anti-mouse IgG secondary antibody (2 mg/mL), (1:20000, Abcam) diluted in blocking buffer.

Protein amount was quantified by using ImageJ software: intensity of the Hsp90 band was normalized to the total protein amount, i.e. the intensity of the entire lane of the Coomassie-stained gel, run simultaneously (described above).

### Evaluation of the mitochondrial morphology and protein import machinery

MitoLoc plasmid has been obtained from Markus Ralser^10^. Each studied yeast strain was transformed according to the described protocol^35^ with the only difference that the cells were incubated with the plasmid overnight at room temperature. Microscope slides were prepared as follows: 150 μL of YPD media containing 2% agarose was placed on a preheated microscope slide, and cooled, before applying yeast cells to obtain a monolayer. The cells were previously centrifuged at 4000 × g for 3 min, and resuspended in 50 μL YPD. Once dry, cover slip was placed and sealed. The slide was mounted on the Volocity software (version 6.3; Perkin Elmer) driven, temperature-controlled Nikon Ti-E Eclipse inverted/UltraVIEW VoX (Perkin Elmer) spinning disc confocal setup. Images were recorded through 60xCFI PlanApo VC oil objective (NA 1.4) using coherent solid state 488 nm and 543 nm diode lasers with DPSS module, and 1000 × 1000 pixels 14 bit Hamamatsu (C9100-50) electron-multiplied, charge-coupled device (EMCCD). The exposure time was 100 ms for GFP and 300 ms for mCherry, and 5–10% laser intensity was used. The number of cells with cytosolic mCherry accumulation was counted manually. Approximately 1000 cells of each strain were examined. Images were analysed using ImageJ software with the MitoLoc plugin.

### Blue Native-PAGE

Mitochondria were isolated as previously described^36^. Mitochondria were solubilized in 1% digitonin, 20 mM Tris/HCl (pH 7.4), 5 mM EDTA, 100 mM NaCl, 10% (w/v) glycerol, and 2 mM PMSF to a final concentration of 1 mg/mL for 30 min at 4 °C. Lysates were cleared by centrifugation (20000 × g, 15 min, 4 °C) before addition of 10x loading dye (5% Coomassie brilliant blue G-250, 500 mM 6-aminohexanoic acid, 100 mM Bis-Tris, pH 7.0) and separated on 4–13% polyacrylamide gradient gels with 4% stacking gel, as described^37^. It should be noted that during the representative experiment we have skipped two lanes between the final two samples due to less than perfect well shape in those lanes. However, all displayed lanes are from the same gel.

For the Western blot determination of respiratory chain components, mitochondrial proteins were isolated from solubilized mitochondria and separated by using SDS-PAGE electrophoresis. After transfer, the membrane was decorated with antibodies against Qcr8, Cox5, Cox15, Coa3, Atp5, Tim21, Tim23, Tom40 and Porin. The experiment was repeated three times. The image in Fig. 2 displays a representative result while the quantification and statistics was done on all replicates. The signal was quantified by using ImageJ software: the intensity of the band of each protein was normalized to the intensity of porin, used as a loading control.

### ATP level and NADP/NADPH ratio measurement

ATP Colorimetric/Fluorometric Assay Kit (BioVision) was used to measure ATP levels in all strains. This method utilizes glycerol phosphorylation to generate a product easily quantifiable at 570 nm. 2 × 10^6^ cells were lysed in 100 μL ATP Assay Buffer in the presence of zymolyase (0.1 U/μL) and incubated for 1 hr at 37 °C. Protein concentration was measured with Bradford reagent (Sigma). Cell lysate was treated with Deproteinization Sample Preparation Kit (BioVision, Cat. #1997), based on PCA protein precipitation method. Absorbance was measured at 570 nm in a micro-plate reader. The amounts of ATP in the samples were calculated according to the standard curve and normalized to total protein concentration.

NADP/NADPH ratio was measured using NADP/NADPH quantitation kit (Sigma-Aldrich) according to the manufacturers instructions.

### RNA extraction

Total RNA was isolated from yeast cells following the procedure of the NucleoSpin RNA kit (Macherey&Nagel) for up to 3 × 10^8^ yeast cells, which dictates incubation with 50–100 U of zymolyase for 1 hr at 30 °C. The quality of resulting total mRNA was tested on 1% agarose gels.

### Quantitative real-time PCR

cDNA was synthesized from 1000 ng of total RNA using iScript^TM^ cDNA Synthesis Kit (Biorad). The resulting cDNA was diluted 100x, mixed with primer pairs for each gene and SYBRgreen (BioRad). All primer pairs were designed to have a melting temperature of 60 °C and are listed in Supplementary Table 1. The qPCR reaction was run on a QuantFlexStudio 6 (Life Technologies) using 40 cycles, after which the melting curves for each well were determined. Final fold change values were estimated relative to the UBC6 gene in the control strain replicates.

### Statistical analysis

Statistical analysis of data was performed using R v2.15.3 (*CRAN,* http://cran.r-project.org) and RStudio for Windows, v 0.97 (http://www.rstudio.com/). All groups were tested for normality of distribution using Shapiro-Wilk test. Since data followed normal distribution, the differences between multiple groups were compared using parametric one-way ANOVA, followed by Tukey’s *post-hoc* test. The differences between two groups were tested using Student’s two-tailed t-test. For all tests significance level was set at p < 0.05.

## Additional Information

**How to cite this article**: Perić, M. *et al*. Crosstalk between cellular compartments protects against proteotoxicity and extends lifespan. *Sci. Rep.* **6**, 28751; doi: 10.1038/srep28751 (2016).

## Supplementary Material

Supplementary Information
