# Supplementary material for: Crosstalk between cellular compartments protects against proteotoxicity and extends lifespan
Source: Sci Rep. 2016 Jun 27;6:28751. doi: 10.1038/srep28751 (PMC4921836; doi:10.1038/srep28751)
Supplement: Supplementary Information [file srep28751-s1.pdf]

Crosstalk between cellular compartments protects against proteotoxicity and extends lifespan

Matea Perić[1], Peter Bou Dib[2], Sven Dennerlein[2], Marina Musa[1], Marina Rudan[1], Anita Lovrić[1], Andrea Nikolić[1], Ana Šarić[3], Sandra Sobočanec[3], Željka Mačak[3], Nuno Raimundo[2], Anita Kriško[1,*]

[1] Mediterranean Institute for Life Sciences – MedILS, Meštrovićevo šetalište 45, 21000 Split, Croatia
[2] Universitätsmedizin Göttingen, Institut für Zellbiochemie, Humboldtallee 23, D-37073 Göttingen
[3] Division of Molecular Medicine, Ruđer Bošković Institute, Bijenička 54, 10000 Zagreb, Croatia

* corresponding author

Supplementary Figures

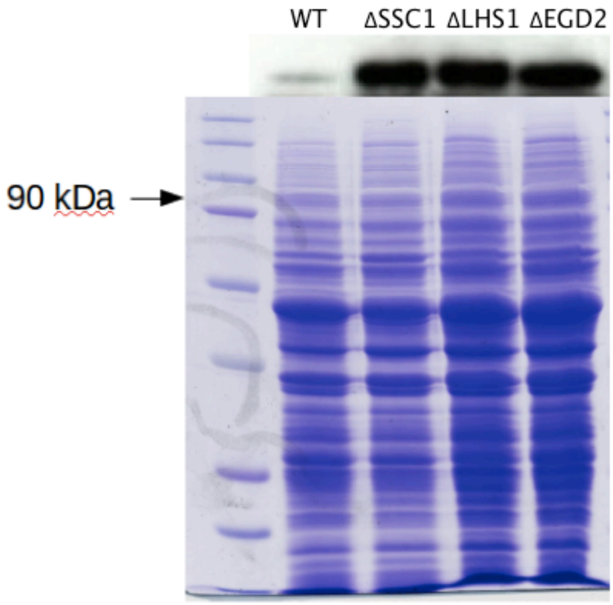

Figure S1. Western blot analysis of the yeast Hsp90 level in the chaperone deficient strains, compared to the WT. Hsp90 level was normalized to total protein level in respective extract. The image displays a representative result of three repetitions of the experiment.

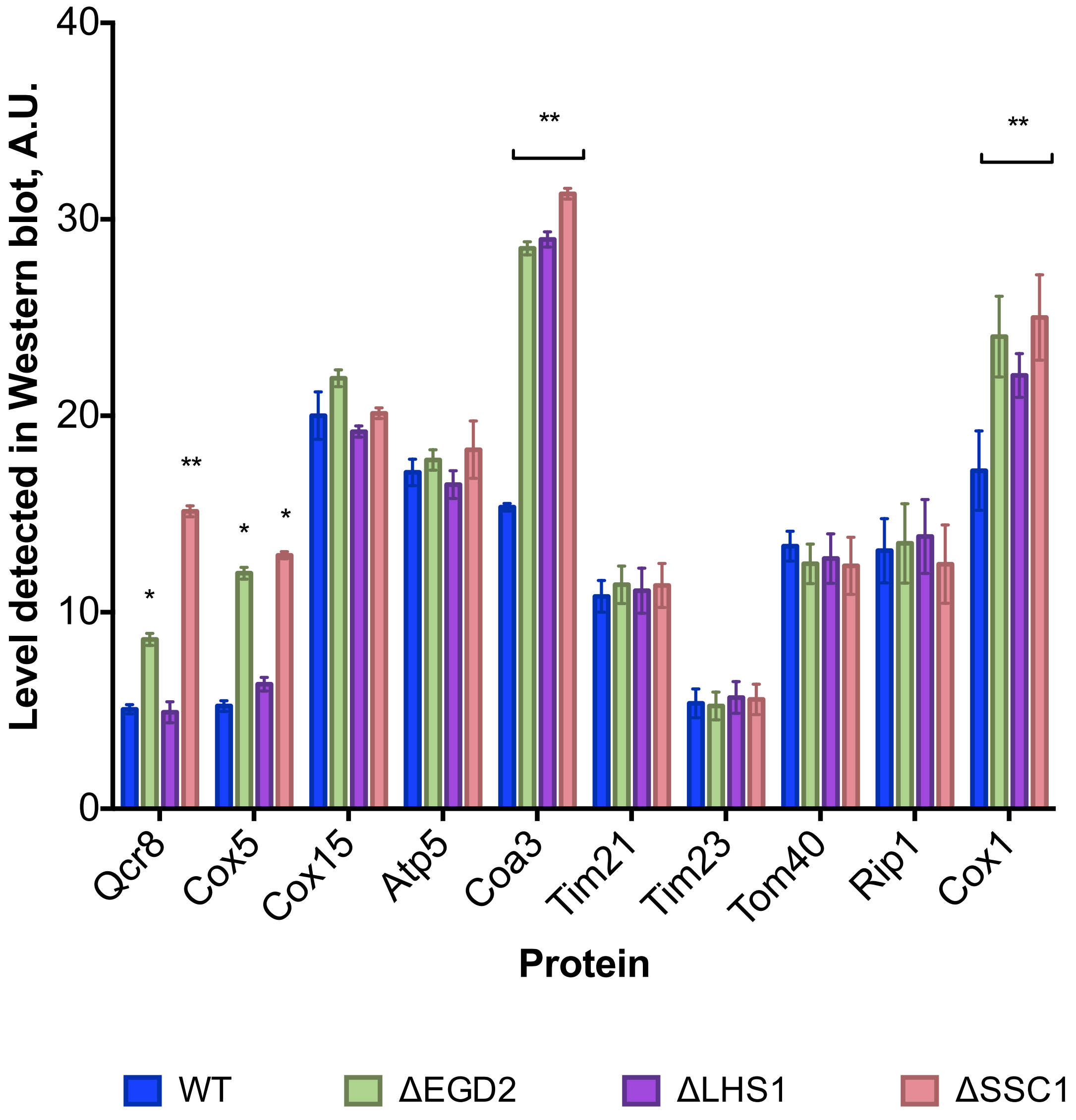

Figure S2. The quantification of the Western blot analysis of the respiratory chain components in the chaperone deficient strains, relative to the WT. Levels of each component were normalized to the intensity of porin which was used as a loading control. *** $p < 0.001$; ** $p < 0.01$; * $p < 0.05$ (ANOVA plus post hoc).

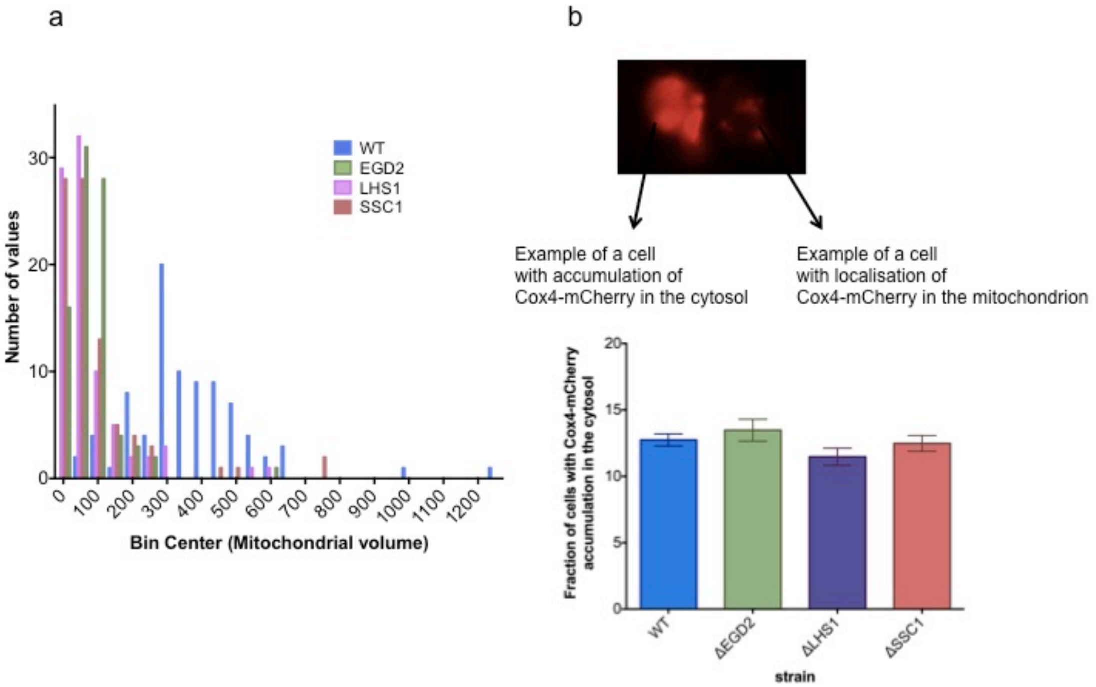

Figure S3. (a) Chaperone deficient strains are characterized by fragmented mitochondria of decreased volume, as quantified by using the MitoLoc plugin for Image J. **(b)** Fraction of cells with Cox4-mCherry remains unchanged in the chaperone deficient strains. Examples of cells with Cox4-mCherry accumulation in the cytosol, as well as those localized in the mitochondria are shown in the top panel. Data are represented as mean ± SD from 3 independent cultures, each measured in duplicate. *** p<0.001; ** p<0.01 (ANOVA plus post hoc).

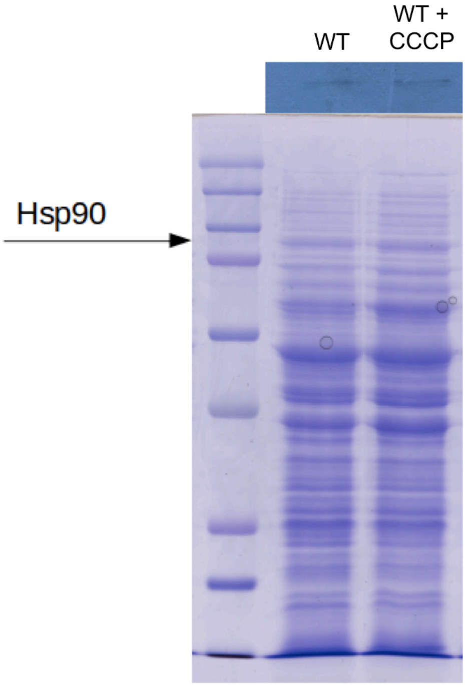

Figure S4. Western blot analysis of the yeast Hsp90 level in the WT strain exposed to 25 μM CCCP, compared to the WT. Hsp90 level was normalized to total protein level in respective extract. The image displays a representative result of three repetitions of the experiment.

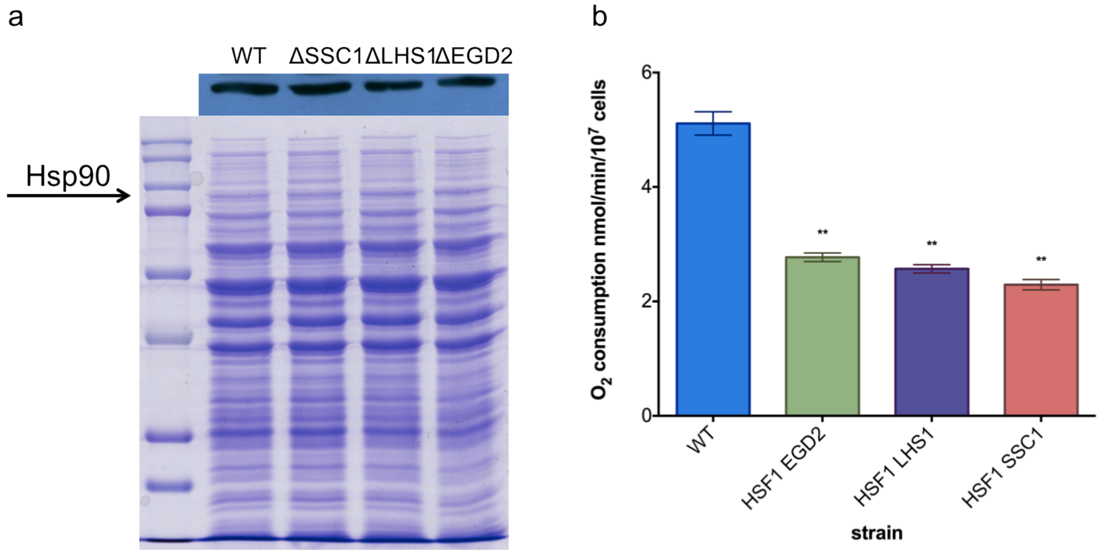

Figure S5. (a) Western blot analysis of the yeast Hsp90 level in the HSF1 and chaperone double deletion strains, compared to the WT. Hsp90 level was normalized to total protein level in respective extract. The image displays a representative result of three repetitions of the experiment. **(b)** Oxygen consumption rates remain decreased in the HSF1-chaperone double deletion strains. Oxygen consumption is measured polarographically in the YPD growth medium supplemented with 2% glucose at 30°C. Data are represented as mean ± SD from 3 independent cultures, each measured in duplicate. *** $p<0.001$; ** $p<0.01$ (ANOVA plus post hoc).

Supplementary Table 1. The list of primers used for qPCR in this study.

HSP26-F	ATG TCA TTT AAC AGT CCA TTT TTT G
HSP26-R	TTA GTT ACC CCA CGA TTC TTG A
HSP42-F	ATG AGT TTT TAT CAA CCA TCC CTA T
HSP42-R	TCA ATT TTC TAC CGT AGG GTT GA
SSA1-F	ATG TCA AAA GCT GTC GGT ATT G
SSA1-R	TTA ATC AAC TTC TTC AAC GGT TG
UBC6-F	ATG TCT AGG GCT AAG AGA ATT AT
UBC6-R	TCA CTT CAA CAA TTC CTC GAT G
SOD1-F	ATG GTT CAA GCA GTC GCA GTG TTA AA
SOD1-R	TTG AAA GGA TTG AAG TGA GGA CCA G
CIT2-F	ATG ACA GTT CCT TAT CTA AAT TCA AAC AGA A
CIT2-R	TCC CTG GAA TAC CTC TCA TAC CAC

CIT1-F	ATG TCA GCG ATA TTA TCA ACA ACT AGC AA
CIT1-R	GGT TTT ACC GTG TTC TTT CTT GAA TTT TTT A
IDH1-F	GCT TAA CAG AAC AAT TGC TAA GAG
IDH1-R	GTT GAT GAT TTC ATT CGT GAA GTC
HSP60-F	ATG TTG AGA TCA TCC GTT GTT C
HSP60-R	TTA CAT CAT ACC TGG CAT TCC T
PIM1-F	ATG CTA AGA ACA AGA ACC ACA AAG ACC
PIM1-R	TTC AAT TGA TGC TGG AAC TCT TGC CA
MCX1-F	ATG TTG AAA TCT GCA AGC CAA AA
MCX1-R	TTA TGT TAA CGT TCT CTT GGG AA
SOD2-F	ATG TTC GCG AAA ACA GCA GCT GC
SOD2-R	ATT GGT CAA CAG CAG TGT TGA ATC C
COX1-F	ATG GTA CAA AGA TGA TTA TAT TCA ACA AAT GC
COX1-R	ATT AAA GCA GGC ATT ACT AAG AAG AAA ATC A
COX3-F	ATG ACA CAT TTA GAA AGA AGT AGA CAT CAA C
COX3-R	AT GTA GCT TCA GCT ACA ATA TCT CTA AAT C
COX4-F	ATG CTT TCA CTA CGT CAA TCT ATA AGA TTT TT
COX4-R	TCT AAC CTA GCT AAA CCA GTT TCT TGA T
KAR2-F	GTT TTT CAA CAG ACT AAG CGC T
KAR2-R	CTA CAA TTC GTC GTG TTC GAA A
PDI1-F	ATG AAG TTT TCT GCT GGT GCC G
PDI1-R	TTA CAA TTC ATC GTG AAT GGC ATC
SEC62-F	ATG TCA GCC GTA GGT CCA G
SEC62-R	GGC TTT TTC ATT GAT GGC TTT C
DER1-F	ATG GAT GCT GTA ATA CTG AAT CTC
DER1-R	ATG CTG GAT AAA TTT ATC TGG TAT
HRD1-F	ATG GTG CCA GAA AAT AGA AGG A
HRD1-R	ATG CTG GAT AAA TTT ATC TGG TAT
